# Supplementary material for: The Prevalence and Determinants of Hesitancy for Regular COVID-19 Vaccination among Primary Healthcare Patients with Asthma or COPD in Greece: A Cross-Sectional Study
Source: Vaccines (Basel). 2024 Apr 14;12(4):414. doi: 10.3390/vaccines12040414 (PMC11054093; doi:10.3390/vaccines12040414)
Supplement: Supplementary file 1 [file vaccines-12-00414-s001.zip › vaccines-2944736-supplementary.pdf]

ATTITUDES AND PERCEPTIONS ABOUT THE COVID-19 VACCINE IN PATIENTS WITH COPD/ASTHMA

1.	**Full name (initials):**			
2.	**Residence:**			
3.	**Gender:**	Male ☐	Female ☐	
4.	**Age:**			
5.	**Smoking:**	Yes ☐	No ☐	Ex-smoker ☐
6.	**Education:**			
7.	**Marital status:**			
8.	**General health status:**	Good ☐	Moderate ☐	Bad ☐
9.	**Chronic Conditions:**			
10.	**Medication:**			
11.	**Do you belong to a vulnerable population?**	YES ☐	NO ☐	
12.	**Do people who live with you belong to a vulnerable population?**	YES ☐	NO ☐	
13.	**COVID vaccination:**	YES ☐	NO ☐	
14.	**When?**			
15.	**Number of doses:**			
16.	**Side effects?**	Mild ☐	Moderate ☐	Severe ☐
17.	**Have you ever been infected by COVID-19:**	YES ☐	NO ☐	
18.	**When?**			
19.	**How would you describe your disease?**	Mild ☐	Moderate ☐	Severe ☐
20.	**If the COVID-19 vaccine had to be given regularly every year, will you do it?"**	Yes ☐	No ☐	I'm not sure ☐

21. Why would you get a booster shot of the vaccine?

A) My own initiative	B) My doctor recommended it	C) Fear of serious illness
D) Free supply	E) Due to profession	F) Media campaigns
G) Other reasons............		

22. Why wouldn't you get a repeat dose of the vaccine?

A) Insufficient Information	B) Fear of side effects	C) Reported side effects in family/social environment
D) It's part of a conspiracy	E) Doubt of efficacy (does not protect 100%)	F) Not sufficiently tested
G) I have had other vaccines that protect me	H) Due to pregnancy	I) I have been infected by COVID-19, so I don't need it
K) I prefer to get sick	L) I believe that I will not get sick or that even if I get sick it will not be serious	M) I follow my family's point of view
N) Commercial reasons promote the vaccine	O) I don't believe in vaccination in general	P) Other reasons:............................

23. What does affect your opinion on whether to take the booster shot?

A) Religion	B) The political leadership	C) The opinion of scientists
D) Media and the Internet	E) The anti-vaccination movement	F) Worried about getting infected with COVID-19?
G) Would you – Have you had the Flu vaccine/shot?		
